# Repositioning of Fluoroquinolones: A New Approach in Antitumor Therapy

**DOI:** 10.3390/biology15090717

**Published:** 2026-04-30

**Authors:** Jeferson Ricardo da Silva, Jaqueline Barbosa de Souza, Lara Limeira de Oliveira, Luís André de Almeida Campos, Isabella Macário Ferro Cavalcanti

**Affiliations:** 1 Keizo Asami Institute (iLIKA), Federal University of Pernambuco (UFPE), Recife 50670-901, PE, Brazil; jeferson.ricardo@ufpe.br (J.R.d.S.); jaqueline.bsouza@ufpe.br (J.B.d.S.); lara.limeira@ufpe.br (L.L.d.O.); 2 Campus Ouricuri, University of Pernambuco (UPE), Ouricuri 56200-000, PE, Brazil; 3 Academic Center of Vitória (CAV), Federal University of Pernambuco (UFPE), Vitória de Santo Antão 55608-680, PE, Brazil

**Keywords:** oncology, quinolones, antitumor activity

## Abstract

Fluoroquinolones are known for their antibacterial properties, but recent research has revealed their potential to inhibit the growth and proliferation of cancer cells. Thus, this study aimed to emphasize the repositioning and potential of fluoroquinolones as possible therapeutic tools in the fight against cancer, opening up new perspectives for the field of oncology. Thus, this paper consists of a descriptive literature review of recent studies published between 2013 and 2023 on the use of fluoroquinolones in anticancer therapy. The results indicate that fluoroquinolones can interfere with the cell cycle, induce apoptosis and oxidative stress, and impact factors associated with tumorigenesis, such as the transcription of ectopic expression of SNAI1 for epithelial–mesenchymal transition. In addition, studies show that combining fluoroquinolones with other antineoplastic agents can increase their efficacy, and the possibility of encapsulating these drugs in controlled-release systems is also emerging as a promising antitumor strategy. In conclusion, repositioning fluoroquinolones in antitumor therapy presents an expanding field of research, offering new perspectives for cancer treatment. However, more studies are needed to fully elucidate their potential and establish effective clinical use protocols.

## Simple Summary

Studies indicate that, in addition to their well-known antibacterial properties, fluoroquinolones also show potential in cancer treatment. This work, through a descriptive literature review conducted between 2013 and 2025, highlights the possibility of repositioning these drugs as anticancer therapeutic agents. Evidence suggests that fluoroquinolones can interfere with the cell cycle, induce apoptosis and oxidative stress, and impact factors related to tumorigenesis, such as the expression of the *SNAI1* gene. Additionally, the combination of fluoroquinolones with other antineoplastic agents has been shown to enhance therapeutic efficacy, and the encapsulation of these substances in controlled-release systems emerges as a promising strategy. It is concluded that the repositioning of fluoroquinolones for use in antitumor therapy represents a growing field, with the potential to innovate cancer treatment. However, further studies are needed to fully understand their mechanisms of action and to develop effective clinical protocols.

## 1. Introduction

Cancer remains a global health challenge and is a focal point in therapeutic research [1,2]. Studies highlight the importance of mutations in crucial factors such as genomic instability, immune evasion, tumor microenvironment, and induced angiogenesis. Thus, the multifactorial etiology of cancer results from complex interactions between genetic, environmental, and lifestyle factors. In 2020, it was reported that there were about 19.3 million new cancer cases, resulting in approximately 10 million related deaths worldwide [3,4].

In terms of treatment, there are numerous conventional approaches to cancer. These include chemotherapy, radiation therapy, surgery, and immunotherapy. These aim to eliminate cancerous cells or inhibit their growth. However, they present significant challenges, including an increased risk of recurrence and toxicity. In addition to conventional therapies, other treatment modalities such as hormone therapy (e.g., in breast and prostate cancers), targeted therapies, and precision medicine approaches have become increasingly relevant, contributing to improved patient stratification and therapeutic outcomes [5,6].

In this context, drug repositioning emerges as a promising therapeutic approach in which already approved drugs are used for new therapeutic indications different from their original use [7,8]. This approach offers several advantages, including high marketability and a lower probability of failure. Moreover, it offers an economical alternative to new drug development by reducing the time and costs associated with regulatory approval. Additionally, it demonstrates significant potential for rare diseases, opening doors to multiple novel therapeutic opportunities [9].

Facing this context, the reuse of antibiotics emerges as a proposal in cancer therapy. Although traditionally those are employed in bacterial infections, recent research emphasizes the possibility of antibiotic repositioning for oncological treatment [10,11].

Among the antibiotics that can be used for this purpose, fluoroquinolones (FQs) have been attracting interest as potential agents in tumor cell treatment. A common example is ciprofloxacin (CIP), which stands out for its cytotoxicity in various cancer cell lineages through mechanisms that interrupt DNA replication processes, leading to cell cycle arrest and apoptosis [12]. This versatility enables that, possibly, drugs from this class can either act in an isolated way or in a synergistic way with other anticancer drugs, leading to cell death [10,13].

Therefore, this study aims to examine the potential of fluoroquinolones as therapeutic candidates in oncology, contributing to ongoing discussions on drug repositioning strategies. Recent advances published between 2023 and 2025 have expanded the current understanding of drug repurposing in cancer, supporting further investigation of fluoroquinolones in this context. In contrast to previous reviews, this work seeks to integrate mechanistic insights with translational limitations, offering a more critical and balanced perspective on the potential repositioning of fluoroquinolones in oncology.

## 2. Cancer

Cancer comprises a heterogeneous group of diseases characterized by uncontrolled cell proliferation and diverse molecular alterations. Its complexity arises from variations in genetic mutations, tissue origin, and tumor microenvironment, which influence disease progression and therapeutic response. However, the variation in cancer incidence patterns among different age groups and human tissues represents a substantial challenge to therapeutic development [1,5].

Given this panorama, it is foreseeable that the number of patients affected by the disease will continue to grow globally in the next five decades. Demographic changes, including aging and population growth, strongly influence cancer incidence trends, which vary among regions [14,15,16,17,18].

Given that cancer is defined by the uncontrolled proliferation of abnormal cells, it presents a surprising diversity of forms, each with its own molecular features and unique therapeutic options. Therefore, it is necessary to devise innovative approaches beyond conventional therapies. In this context, it is considered that the development of more targeted and effective therapeutic strategies is crucial to improve the clinical outcomes and quality of life for individuals afflicted by this high-mortality and high-morbidity disease [19,20].

In the present era, a variety of conventional treatments are available to combat cancer; these include chemotherapy, radiation therapy, surgery, and the recently developed immunotherapy. These approaches aim to eliminate malignant cells or inhibit their growth [21]. Despite the advances that have increased the life expectancy of patients undergoing this type of therapy, it is well documented that persistent recurrence results in a short survival rate. In the face of this reality, it is necessary to develop novel therapeutic strategies across the various phases of cancer, whether in its initial stage, in cases of metastasis, or in the occurrence of relapses. Therefore, drug repositioning presents hope to enhance the fight against cancer [18,22].

## 3. Drug Repositioning

The pharmaceutical industry faces a significant challenge in the development of new drugs, primarily because this process requires an approximate period of 12 to 15 years and substantial costs, totaling around 2 billion dollars per drug. This process encompasses several stages, including pre-clinical investigations, clinical trials, and post-release monitoring to ensure safety. Furthermore, the success rate is extremely limited, with only 2% of drugs achieving approval [23,24].

Despite recent significant investments in developing new medications, only 22 newly created drugs received approval between 2014 and 2016. This sharply contrasts with the 53 drugs approved two decades earlier, highlighting a considerable decrease in the approval rate. Given the current circumstances, a promising approach to address this issue is the strategy of drug repositioning [25,26].

The repositioning of drugs, also referred to as drug reprofiling, is defined as the process in which already identified drugs are used for new therapeutic indications other than those for which they were originally designed [27]. This approach presents a series of benefits, leading numerous biotechnology and pharmaceutical companies to target this strategy due to its highly profitable nature, low failure rates, and reduced costs, since it is an already approved medicine. Consequently, this approach also requires a shorter time frame for its release and, especially, offers therapeutic opportunities for rare diseases [9,28].

This is attributable to the fact that repurposed medicines have already undergone a series of short- and long-term toxicity tests, as well as pharmacokinetic and pharmacodynamic studies (PK/PD). Based on this established foundation, these drugs can be promptly sent to clinical trials in a reduced timeframe. If regulatory approval is obtained, their integration into clinical practice is typically accelerated once the repositioned drugs are approved in a shorter period (3 to 12 years) compared to conventional development [29].

Given the importance of cancer to public health, the antibiotics and drugs that are already present in the market and are used in clinical practice have been investigated with the purpose of acting in tumor inhibition/eradication through mechanisms that involve the phosphorylation of the ribosomal protein p70 S6 kinase (azithromycin) to the induction of apoptosis through the inhibition of the eIF4E-β-catenin axis (nifuroxazide). Therefore, as the necessity for novel therapeutic approaches in cancer treatment continues to increase, drug repositioning emerges as a strategy that can offer results more efficiently in terms of time and cost, representing a promising approach to fight this disease [30,31].

## 4. Antibiotics with Antitumor Potential

Antibiotics, regardless of their natural or synthetic origin, act in two distinct ways: by interrupting bacterial growth or by causing direct bacterial death. Bactericidal antibiotics have the effect of causing bacterial death, while bacteriostatic antibiotics act by preventing bacterial growth [32]. Despite their general application in the treatment of bacterial infections, recent investigations have suggested the viability of reusing antibiotics for therapies against cancer [33].

The intersection between cancer therapy and the use of antibiotics remains a complex issue. Studies have presented unfavorable clinical results for individuals combining immune checkpoint inhibitors (ICIs) with antibiotics [34]. Recently, research has highlighted the importance of a careful investigation into anti-PD-1/PD-L1 treatment in patients with cancer who are also under antibiotics. This evaluation is critical to mitigate the potential risks of immune system-related side effects. Several studies have investigated the impact of antibiotics on the efficacy of immune checkpoint inhibitors, including anti-PD-1 and anti-PD-L1 therapies. Broad-spectrum antibiotics, such as β-lactams, fluoroquinolones, and macrolides, have been associated with alterations in gut microbiota composition, which may impair immunotherapy response. For instance, disruption of commensal bacterial populations has been linked to reduced T-cell activation and poorer outcomes in patients receiving PD-1/PD-L1 inhibitors [33,35].

In the present scenario, numerous studies have demonstrated the potential of antibiotics to be employed in an efficient manner in the targeted therapy of cancer. A notable example is the evaluation conducted by scientists on the antibiotic trimethoprim, which demonstrated the ability to reduce the levels of the Snail protein in human colorectal cancer cells, in addition to impacting both human and murine breast cancer cells. In another study, it was observed that this antibiotic could stimulate the activity of caspases 3, 8, and 9, which are related to melanoma skin cancer [10,36].

Another promising approach involves the combination of doxycycline, azithromycin, and vitamin C, which specifically targets cancer stem cells through a “synthetic–metabolic” strategy, an approach that aims to eliminate the cancerous stem cells by targeting their mitochondria. This focus has shown great potential in combating these stem cells, making this strategy a highly promising alternative [37].

Recent investigations have highlighted the antitumor properties of FQs. Remarkably, both ciprofloxacin and moxifloxacin have demonstrated antiproliferative and pro-apoptotic effects in various cancer cell lines, including prostate, colon, breast, and lung adenocarcinomas [38,39,40,41].

## 5. Fluoroquinolones

FQs represent a class of synthetic antibiotics whose clinical value was discovered in 1962 during the analogous synthesis of a leader structure: 7-chloro-1-ethyl-1,4-dihydro-4-oxo-3-quinolinecarboxylic acid. This synthesis, originally aimed at producing chloroquine (an antimalarial agent), generated a byproduct derived from the original molecule, known as nalidixic acid [41,42]. Among the resulting compounds of this process, nalidixic acid has stood out due to its moderate activity against Gram-negative bacterial strains. Consequently, nalidixic acid was introduced into clinical practice in 1964, even though its use was initially restricted in the treatment of urinary tract infections. Although quinolones originated as byproducts during chloroquine synthesis, their mechanism of action differs significantly from that of classical antimalarial drugs. While antimalarials such as chloroquine act primarily by interfering with heme detoxification in *Plasmodium* parasites, fluoroquinolones exert antibacterial effects by inhibiting DNA gyrase and topoisomerase IV. Thus, their biological activity as antimicrobial agents is mechanistically distinct from antimalarial compounds [43].

However, a timeframe of 10 years of research was necessary to develop the first generation of fluoroquinolones, which was able to surpass the limitations of the original compound. These limitations included low bioavailability and an exclusive affinity to Gram-negative bacteria. During this timeframe, the progress of investigations led to refining the properties of FQs and overcoming their initial restrictions. Structural modifications in fluoroquinolones, particularly at positions N-1, C-7, and C-8, have been associated with variations in biological activity. These changes may influence not only antimicrobial potency but also cellular uptake, DNA binding capacity, and potential anticancer effects, highlighting the importance of structure–activity relationships in drug repositioning [44,45].

Quinolones were categorized into four generations based on their structure and mechanism of action. In the first-generation quinolone category, nalidixic acid occupied a leading position. However, the specific addition of a fluorine atom in the R-6 position and the alteration of the quinolone ring resulted in the creation of the current FQ, which demonstrates notable efficiency against Gram-negative, Gram-positive, and anaerobic bacteria [46,47].

The second generation comprises various members, such as ciprofloxacin, norfloxacin, lomefloxacin, ofloxacin, and enoxacin. Among them, ciprofloxacin is the most widely prescribed due to its efficiency in treating a wide range of infections. Meanwhile, norfloxacin occupies the second position, even though its use is limited to treating specific infections due to its limited tissue penetration, despite being the pioneer among wide-spectrum antibiotics [48,49].

Third-generation FQs include levofloxacin, clinafloxacin, grepafloxacin, and sparfloxacin, as well as other variations, and they are notable for their extended efficacy against Gram-positive bacteria. Moreover, these FQs also offer broad coverage against Gram-negative bacteria such as trovafloxacin, gatifloxacin, and moxifloxacin [45].

The fourth generation of fluoroquinolones includes compounds such as delafloxacin, finafloxacin, and zabofloxacin. These agents are characterized by enhanced activity against Gram-positive bacteria, including resistant strains, as well as improved efficacy in acidic environments. Structurally, modifications in their chemical backbone contribute to increased cellular penetration and a broader antimicrobial spectrum. Although their antibacterial properties are well established, their potential anticancer effects remain underexplored and warrant further investigation [46,48].

FQs’ mechanism of action is closely related to the inhibition of enzymes responsible for controlling bacterial DNA, DNA gyrase, and topoisomerase IV. In this sense, they interfere with the processes of replication, transcription, and bacterial DNA repair, resulting in the inhibition of bacterial growth and, eventually, in cell death [49].

**Figure 1:**
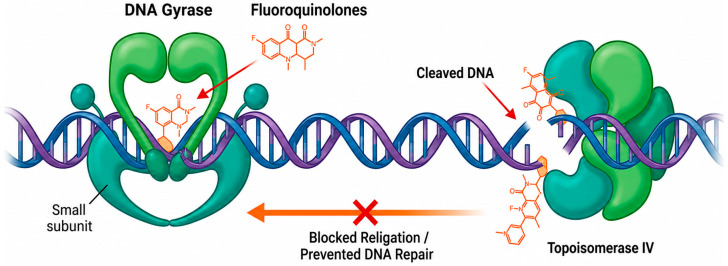
Mechanism of action of FQs against DNA gyrase and topoisomerase IV. Legend: Fluoroquinolones inhibit DNA supercoiling and relaxation by binding to gyrase and DNA, thereby stabilizing the gyrase–DNA–cleavage complex. In Gram-positive bacteria, topoisomerase IV is the primary target. During replication, gyrase and topoisomerase IV induce double-strand breaks in the DNA to relieve positive supercoiling. This process forms the DNA cleavage complex, which involves the binding of the enzymes to the DNA. Quinolones bind to this enzyme–DNA complex, inhibiting DNA replication and leading to bacterial cell death.

DNA gyrase is an enzyme that performs a crucial role in the regulation of the state of DNA supercoiling in bacteria. FQs interact with subunit A of the DNA gyrase, forming a stable complex of DNA-FQ-DNA gyrase. This complex prevents the DNA gyrase’s ability to relax the supercoiling of the DNA, resulting in damage to the bacterial DNA [50]. Topoisomerase IV is also a crucial enzyme involved in the DNA replication process. FQs, when interacting with topoisomerase IV, interfere with the separation and reconnection of DNA strands during replication, leading to errors in the duplication of bacterial genetic material (Figure 1) [51]. This impairment of the enzymes DNA gyrase and topoisomerase IV by FQs is crucial to the success of the treatment, as it compromises the bacteria’s ability to maintain the integrity of their DNA. This damage is involved in the inhibition of bacterial growth and, eventually, in cell death [48].

## 6. Fluoroquinolones with Antitumor Activity

Substances with anticancer activity are classified as either cytotoxic, inducing cell death, or cytostatic substances, which inhibit cell proliferation, both contributing to the reduction in tumor growth. Among this group of substances, FQs stand out, representing a pharmacological class highly explored and with well-established anticancer properties. In addition to their well-known action on bacterial DNA gyrase and topoisomerase IV, fluoroquinolones may also interact with eukaryotic topoisomerase II. This interaction can lead to DNA damage, cell cycle arrest, and apoptosis in cancer cells. Although the affinity for eukaryotic enzymes is generally lower than for bacterial targets, this mechanism may partially explain the observed antitumor effects of fluoroquinolones [12,52].

The importance of the FQ has been the subject of considerable interest in several recent studies. While the primary action of the quinolones involves the inhibition of bacterial topoisomerase IV, they also exhibit the ability to interfere with analogous enzymes in eukaryotic cells. Those versatile agents are able to induce apoptosis, interrupt the cell cycle, and consequently promote cell death in several cancerous cell lines, acting alone or in combination with other anticancer agents [53]. Table 1 shows studies that have looked into the possible use of FQs against various types of cancer. Several antibiotics from the FQs class exhibit notorious cytotoxicity in various types of cancer cells.

Although Table 1 summarizes multiple studies demonstrating anticancer activity, it is important to note that the predominance of in vitro data limits the interpretation of these results in a clinical context. Variability in experimental design, cell lines, and drug concentrations further complicates direct comparisons between studies [69].

**Figure 2:**
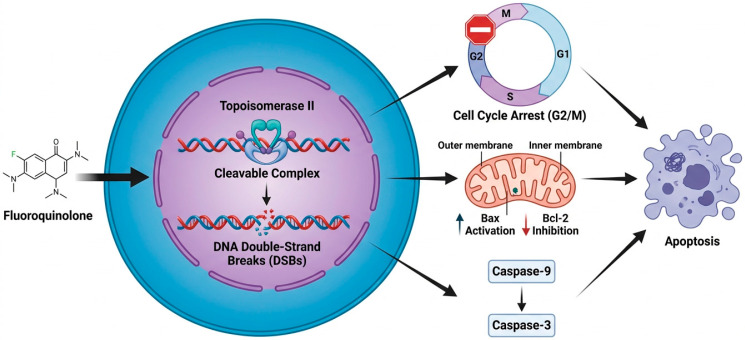
Mechanism of action of the antitumor activity of fluoroquinolones. Legend: The antitumor activity of fluoroquinolones stands out for their minimal side effects and their ability to interfere with DNA synthesis, leading to disruption of the cell cycle. Additionally, they stimulate apoptotic reactions in various cancer cell lines, induce mitochondrial dysfunction, activate caspases-3/9, and promote oxidative stress in cells. Created by BioRender.

CIP stands out for its minimal side effects and for its ability to interfere with DNA synthesis, leading to an interruption in the cell cycle and apoptosis induction (Figure 2). Moreover, evidence shows that CIP, as well as levofloxacin and lomefloxacin, presents antitumor characteristics, stimulating apoptotic reactions in various cancer cell lineages [39]. Cell cycle regulation is crucial for controlling the proliferation, metastasis, and recurrence of cancer cells. This process involves the duplication and segregation of cellular components, preparing them for division. In eukaryotic cells, DNA replication occurs exclusively during the S phase (synthesis), while chromosomal segregation takes place during the M phase (mitosis) [70,71]. The cell cycle is marked by two gap phases: G1 and G2. Notably, CIP can also inhibit the development of colorectal cancer, pancreatic cancer, and breast cancer through its mitochondrial cytotoxicity, underscoring its therapeutic potential in oncology [72].

The relevance of the *p53* gene in inducing apoptosis as a measure of tumor suppression has been widely explored, with an accumulation of evidence validating its role in inhibiting cancer development [53]. This gene is responsible for initiating apoptosis in specific cell types and is frequently the target of mutations in more than 50% of human cancer cases. Mutation of the *p53* tumor suppressor gene paves the way for the activation of pro-apoptotic factors such as Bax while inhibiting anti-apoptotic factors such as Bcl-2, thus facilitating the triggering of apoptosis [73].

In addition to ciprofloxacin, various other FQs have the ability to induce apoptosis, employing different mechanisms. These include a reduction in glutathione levels, induction of mitochondrial dysfunction, increased release of cytochrome c, activation of caspases-3/9, and promotion of oxidative stress in cells [25,62].

An important consideration is whether fluoroquinolones selectively target cancer cells. Although tumor cells are generally more susceptible due to their high proliferation rate and metabolic alterations, evidence suggests that fluoroquinolones may also affect normal cells, particularly at higher concentrations. This raises concerns regarding potential off-target effects and highlights the need for strategies to improve tumor selectivity [62].

Although fluoroquinolones are widely used as antibacterial agents and are generally considered safe, with relatively low cytotoxicity even at high clinical doses (~1000 mg/day), most studies summarized here demonstrate moderate to low anticancer activity, often requiring relatively high concentrations in vitro. This raises important concerns regarding their translational potential, particularly whether effective antitumor concentrations can be achieved in vivo without toxicity [25]. Additionally, there is considerable variability among compounds, cell lines, and experimental conditions, which limits direct comparison across studies. Differences in IC_50_ values may reflect not only intrinsic drug potency but also variations in cellular uptake, metabolic state, and experimental design [73].

Notably, ciprofloxacin appears to exhibit comparatively higher activity, with IC_50_ values as low as 0.97 µM in HCT116 and A549 cells, suggesting that specific structural or pharmacological properties may enhance its anticancer effects. Despite these findings, the predominance of in vitro data and the lack of standardized methodologies highlight the need for more robust in vivo studies and well-designed clinical investigations to better define the therapeutic relevance of fluoroquinolones in oncology [62].

Enoxacin, a second-generation fluoroquinolone (FQ), has demonstrated its ability to activate microRNA (miRNA) biogenesis in cancer cells. Cancer-specific miRNAs can be categorized into two groups based on their target proteins: oncogenes and tumor suppressor miRNAs [74]. Imbalances in miRNA regulation play a role in the transformation of healthy cells into cancerous cells. This observation has led to the development of two anticancer strategies focused on miRNA pathways: inhibiting miRNA function to prevent suppression of tumor suppressor gene mRNAs or enhancing miRNA function to degrade mRNAs encoded by oncogenes. According to Valianatos et al. [75], enoxacin has shown a positive impact on TARBP2-mediated miRNA processing in various cancer cell lines. Moreover, it has demonstrated specific inhibition of cell growth in both in vitro and in vivo assays [60,76].

The Snail transcription factor plays a central role in epithelial–mesenchymal transition (EMT), a process essential for tumor invasion and metastasis. It represses E-cadherin expression and promotes mesenchymal markers such as N-cadherin and vimentin. Fluoroquinolones such as gemifloxacin have been shown to inhibit Snail expression through modulation of NF-κB signaling pathways, thereby suppressing EMT and reducing metastatic potential. This mechanism highlights a non-classical anticancer pathway independent of direct cytotoxicity [63].

The impact of this drug on the migration and invasion of cancer cells is related to the modulation of Snail expression and the inhibition of NF-κB. This affects the ability of cancer cells to undergo epithelial-to-mesenchymal transition. Inhibition of Snail promotes the restoration of E-cadherin protein and control of mesenchymal genetic markers such as N-cadherin and vimentin. Consequently, this scenario contributes to a decrease in metastasis, as the concentration of the Snail gene in cellular nuclei is reduced. These findings underscore the potential of fluoroquinolones, especially gemifloxacin, in cancer treatment [64]. Notably, some studies report variable efficacy depending on the cancer type and experimental conditions, suggesting that the anticancer effects of fluoroquinolones may not be universal and require further investigation.

Despite the encouraging findings, the current body of evidence presents several limitations. Most studies rely heavily on in vitro models, with limited validation in in vivo systems. Additionally, inconsistencies between studies regarding effective concentrations and mechanisms of action highlight the need for standardized experimental approaches. Furthermore, the lack of clinical trials significantly restricts the translational potential of fluoroquinolones in oncology.

### 6.1. FQs in Combined Use in Antitumor Therapy

Available studies have demonstrated that the combination of anticancer drugs can result in a significant increase in their therapeutic efficacy. This phenomenon can be explained by the fact that the co-administered drugs act through distinct mechanisms, leading to increased cancer cell death and reduced chances of developing resistance to treatment. Furthermore, this strategy is designed to mitigate potential toxicity-related issues, and thus, this approach can be implemented in various ways, such as combining one chemotherapeutic agent with another or integrating it with a different compound, thereby expanding the treatment possibilities in the fight against cancer [25,77].

Currently, various strategies for combining FQs with antineoplastic agents are being extensively investigated. Levofloxacin alone has demonstrated efficacy against several cancer cell lines, including breast cancer, lung cancer, and melanoma. Aydemir et al. [77] revealed that the combination of levofloxacin with cisplatin showed promising results in the treatment of cell carcinoma. Over a 24 h period, the effects of levofloxacin, cisplatin, and the levofloxacin–cisplatin combination on SCC-15 cell viability were observed. The results indicated that the viability rates were 90%, 67%, and 80.8%, respectively.

In contrast, doxorubicin, an anthracycline widely used as a chemotherapeutic agent, has exhibited only minimal impact on Ewing’s sarcoma and lung cancer when administered alone. However, when combined with enoxacin, a significant increase in its efficacy was observed. It is worth noting that although doxorubicin can eliminate a substantial portion of tumor cells, the possibility of post-treatment growth persists, leading to recurrences due to the incomplete elimination of cancer cells. This was evidenced by secondary transplantation experiments of tumor cells treated with doxorubicin, which revealed that tumor growth remained unchanged in comparison to untreated cells [78].

Conversely, lower doses of enoxacin, which are typically employed to treat infections, not only depleted cancer stem cells but also suppressed doxorubicin-induced squamous metaplasia in lung epithelial cells. These results highlight the potential of therapeutic combinations involving FQs and antineoplastic agents to address challenges in cancer treatment [79].

Furthermore, there has been a growing interest in exploring the potential of FQs in the form of fluoroquinolone–metal complexes as highly effective anticancer agents. This approach is based on scientific evidence indicating that certain fluoroquinolone–metal complexes exhibit antitumor activity against leukemia and breast cancer cells. Gold and ruthenium, in particular, have demonstrated efficacy against metastatic cancer cells without harming normal cells, acting through various mechanisms of action [80].

Complexes incorporating lomefloxacin metal ions have demonstrated high efficacy in the treatment of breast cancer cells. Similarly, gold complexes combined with norfloxacin, levofloxacin, and sparfloxacin have been evaluated against various tumor cell lines, including murine melanoma, murine lymphoma, and human myeloid leukemia. Notably, these complexes, compared to free ligands, exhibited greater cytotoxic activity and therapeutic efficacy. The enhanced efficacy of fluoroquinolone–metal complexes may be attributed to several factors, including increased cellular uptake, improved stability, and stronger interactions with DNA and intracellular proteins. Metal ions such as gold and ruthenium can facilitate redox reactions, leading to increased reactive oxygen species (ROS) production and oxidative stress. Additionally, these complexes may enhance DNA binding affinity and disrupt mitochondrial function, thereby amplifying cytotoxic effects compared to free fluoroquinolones [81,82].

### 6.2. Nanoencapsulated FQs

A highly promising approach adopted to intensify antitumor activity is the nanoencapsulation of drugs in controlled-release systems (CRSs) [83]. In antitumor therapy, the most commonly used drug CRSs include liposomes, polymeric nanoparticles, micelles, and inorganic nanoparticles [84]. One of the main advantages of this approach is the improvement in bioavailability and biodegradability, as well as the reduction in drug side effects. In addition, this strategy restricts systemic release, prolonging the circulation of the drugs and facilitating their absorption at the tumor site through optimized permeation, which results in sustained antitumor activity [85,86].

In this context, the use of nanoencapsulated controlled-release systems emerges as a highly beneficial and insightful strategy to optimize the efficacy of antitumor therapies, paving the way for significant advances in cancer treatment. The use of FQs in drug delivery systems has been the subject of extensive research for various pathologies due to their notable antibacterial activity. It is important to note that several bacteria are associated with cancer, including *Escherichia coli* and *Fusobacterium nucleatum*, which are related to colorectal cancer, as well as *Chlamydia trachomatis*, which is associated with cervical cancer. These microorganisms contribute to carcinogenesis through multiple mechanisms, including chronic inflammation, production of genotoxins, and modulation of the host immune response. For example, certain strains of *E. coli* produce colibactin, a genotoxin capable of inducing DNA damage, while *F. nucleatum* promotes tumor progression by activating inflammatory signaling pathways and inhibiting antitumor immune responses. Additionally, *C. trachomatis* has been associated with persistent infections that can lead to cellular transformation and increased cancer risk [87,88].

As reported by Sepich-Poore et al. [89], experimental evidence corroborates that bacterial virulence factors contribute to tumorigenesis. Thus, considering that quinolones can block the cell cycle and induce apoptosis, resulting in the inhibition of cell proliferation, the incorporation of quinolones into drug CRS as antitumor agents represents a notable advance in the field of antitumor therapy. Thus, the intersection between fluoroquinolones, controlled-release systems, and the fight against cancer offers an exciting and promising prospect for more effective and targeted therapy, driving the search for innovative strategies in cancer treatment.

It is also important to consider potential risks associated with fluoroquinolone use, including genotoxicity, mitochondrial toxicity, and the development of antibiotic resistance. These factors may limit their long-term application in oncology and should be carefully evaluated in future studies.

## 7. Conclusions

This review highlights the potential of fluoroquinolones as candidates for drug repositioning in anticancer therapy. Their diverse biological activities, particularly their ability to interfere with the cell cycle, induce apoptosis, and modulate cell migration and invasion, support their investigation in oncology. Additionally, their use in combination with conventional chemotherapeutic agents, as well as their incorporation into controlled drug delivery systems, broadens their therapeutic applicability and may contribute to more targeted and effective cancer treatments.

Future investigations should focus on robust in vivo studies and well-structured clinical trials to substantiate the anticancer effects of fluoroquinolones. Further exploration of structure–activity relationships (SARs), along with advances in targeted delivery strategies and combination regimens, will be important. Special emphasis should be placed on cancer types where mitochondrial dysfunction, epithelial–mesenchymal transition (EMT), and microbiome interactions are relevant, such as colorectal, breast, and lung cancers.

Continued research in this field may improve understanding of the underlying mechanisms, support the optimization of therapeutic approaches, and contribute to better clinical outcomes. Fluoroquinolones may represent a complementary option in cancer therapy, warranting further investigation.

## Figures and Tables

**Table 1:** *In vitro* and/or *in vivo* studies demonstrating the efficacy of fluoroquinolones against different types of cancer.

Drug	Mechanism of Action	Cell Line	*In Vivo/In Vitro*	IC_50_	Reference
Ciprofloxacin	apoptosis induction	NCI-H460 (Lung cancer)	*in vitro*	30.2 µM	[54]
Ciprofloxacin	Cell cycle arrest and apoptosis	COLO829 (Melanoma)	*in vitro*	100 µM	[55]
Ciprofloxacin	Induces apoptosis via p53/BAX/BCL-2	MDA-MB-231 (Breast cancer)	*In Vitro*	4.4 μM	[56]
Ciprofloxacin	Cell cycle arrest and apoptosis	HCT116 (colorectal cancer) and A549 (non-small cell lung carcinoma)	*in vitro*	0.97 µM	[53]
Ciprofloxacin	apoptosis induction	HeLa (cervical cancer)	*in vitro*	16.5 µM	[57]
Ciprofloxacin	Induces apoptosis via caspase-3	HOP-92 (Colon cancer)	*in vitro*		[58]
Ciprofloxacin	apoptosis induction	SW480 (primary colon cancer) SW620 (metastatic colon cancer), PC3 (prostate cancer)	*in vitro*	-	[59]
Enoxacin	TRBP- mediated miRNA biogenesis	LNCaP, 22Rv1, VCaP, DU145 and PC-3 (prostate cancer cells)	*in vitro*	124 μM	[60]
Enoxacin	Apoptosis mediated by ROS pathway activation	AsPC1 (Pancreatic cancer)	*in vitro*	-	[61]
Enoxacin	apoptosis induction	PC-3 (prostate adenocarcinoma)	*in vitro* and *in vivo*	20.2 ± 2.3 − 176.6 ± 16.1 μM	[62]
Gemifloxacin	Reduction in Snail concentration—EMT	SW620 and LoVo (colon cancer)	*in vitro*	51.4 µM	[63]
Gemifloxacin	Reduction in Snail concentration—EMT	MDA-MB-231 and MDA-MB-453 (Human breast adenocarcinoma)	*in vitro* and *in vivo*	51.4 µM	[64]
Levofloxacin	Apoptosis/Inhibition of cell proliferation/Induction of mitochondrial dysfunction and oxidative stress	A549, H3255, NCL-69 and H460 (Lung cancer)	*in vitro* and *in vivo*	-	[65]
Levofloxacin	Inhibition of mitochondrial biogenesis	MCF-7, MDA-MB-231, MDA-MB-468, and SkBr (Breast Cancer)	*in vitro* and *in vivo*	-	[66]
Lomefloxacin	Apoptosis and Oxidative Stress	COLO829 (Melanoma)	*in vitro*	250 µM	[67]
Lomefloxacin	Apoptosis and Oxidative Stress	HL-60 (promyelocytic leukemia)	*in vitro*	-	[68]

NCI-H460: lung cancer cell line; COLO829: metastatic melanoma cell line; MDA-MB-231: breast adenocarcinoma cell line; HCT116: human colon cancer cell line; A549: human alveolar cell carcinoma; HeLa: cervical cancer cell line; HOP-92: human non-small cell lung adenocarcinoma; SW480: human colon adenocarcinoma primary cell line; SW620: human colon adenocarcinoma metastatic cell line; PC-3: human prostate cancer cell line; LNCaP: human prostate cancer cell line; 22Rv1: human prostate cancer cell line; VcaP; human prostate cancer cell line; DU145: human prostate cancer cell line; AsPC1: pancreatic ductal adenocarcinoma cell line; LoVo: human colon metastatic cancer cell line; MDA-MB-453: human breast cancer cell line; A549: lung adenocarcinoma cancer cell line; H3255: lung resistant cancer cell line; NCL-69: lung adenocarcinoma cancer cell line; MCF-7: human breast cancer cell line; MDA-MB-468: human breast cancer cell line; SkBr: human breast cancer cell line; HL-60: promyeloblast leukemia cell line. All IC_50_ values were converted to µM for standardization using the molecular weight of each compound.

## Data Availability

No new data were created or analyzed in this study. Data sharing is not applicable to this article.
